# Remaining Useful Life Prediction of Airplane Engine Based on Bidirectional Mamba and Causal Discovery

**DOI:** 10.3390/s25113429

**Published:** 2025-05-29

**Authors:** Min Li, Longxia Zhu, Meiling Luo, Ting Ke

**Affiliations:** College of Artificial Intelligence, Tianjin University of Science and Technology, Tianjin 300457, China; lxzhu@mail.tust.edu.cn (L.Z.); luomeiling@mail.tust.edu.cn (M.L.); keting@tust.edu.cn (T.K.)

**Keywords:** remaining useful life prediction, Mamba, state space model, causal discovery

## Abstract

Remaining Useful Life (RUL) plays a critical role in prognostics and health management systems. It helps increase reliability and safety for the equipment used in the modern industry. The new idea proposed is the Mamba deep learning model, which aims to find a good balance between predictive performance and computation cost. This paper presents a multimodal RUL prediction model, Cau–BiMamba–LSTM, using causal discovery, a bidirectional Mamba (BiMamba), attention mechanism, and Long Short-Term Memory (LSTM). The framework utilizes maximum information transfer entropy and simple exponential smoothing in building a causal graph model that extracts groups of feature variable groupsLSTM performs long-range dependencies; the attention mechanism dynamically focuses attention according to the temporal context; finally, the bidirectional state space model captures all contextual information over time for a richer insight into underlying data patterns. Tests conducted on the C-MAPSS dataset confirm that this model achieves superior predictive accuracy and robustness. Moreover, the model achieves high predictive performance in very complex, long time–series and provides fast responses.

## 1. Introduction

In the age of Industry 4.0, the basic processes for monitoring and assuring the quality of fault risk management and reliability assessment are essential in maintaining the stability and intactness of operations for industrial equipment. The model for equipment maintenance is changing from passive maintenance to digital precision forecasting, performed through the detection of any faults with high-grade precision in a complex condition of operations [1]; this subsequently reduces the impact of equipment failure on the production process. The recent developments in enhancing GPU logic for deeper learning algorithms speed up the study of good artificial-intelligence-based models for fault prediction and health diagnosis. Accurate prediction of RUL and health status prediction of the equipment enhances the long-term stability of the equipment and reduces maintenance costs. Thus, RUL prediction has become one of the most important directions for research in the field of fault diagnosis and health management.

Current research methods for RUL prediction can be classified broadly into two groups: physics-based methods [2] and data-driven methods [3,4,5,6]. Physics-based methodologies take into account the physical laws and mathematical structures of equipment that explain the physical processes and mechanisms of failure in a reasonable way. However, complex nonlinear relationships and large uncertainties are very often involved in the failure processes of equipment, meaning that those mechanisms are at least well understood, aiding their explanation. In addition, their accuracy heavily relies on the passable estimates of system parameters; even with minor errors in the true parameters, the predictions could significantly deviate from the optimal outcome [7]. They also encounter great difficulties in tackling sudden or stochastic failings. On the other hand, data-driven RUL prediction methods automatically identify nonlinear dependencies associated with the equipment defect process, with minimal reliance on physical parameters. These methods can be broadly applied to various types of equipment. In recent years, data-driven RUL prediction methods based on deep learning have proved to be of incredible potential, with studies being published that include the use of CNNs [8,9], Long Short-Term Memory (LSTM) networks [10], Generative Adversarial Networks (GANs) [11], Graph Neural Networks (GNNs) [12,13,14], attention mechanisms (AMs) [15,16], and Transformer models [17,18]. Keshun et al. [19] proposed an RUL prediction model based on a three-dimensional attention mechanism, CNN, and BiLSTM that enhances prediction accuracy while achieving interpretability. Shi et al. [5] introduced a lightweight novel RUL prediction model integrating exponential smoothing, attention mechanisms, and LSTMs. Such models can be properly exploited in fault diagnosis or health prediction applications which demand quick responses. However, the performance of existing hybrid models using attention mechanisms and classical neural networks typically degrades with longer sequences.

State space models (SSMs) are mathematical representations derived from control systems theory, which describe how states evolve in dynamic systems and define the relationships between inputs and outputs. SSMs rely mainly on their state equations and representation output to use hidden states to preserve historical information [20]. In addition, by alleviating the vanishing gradient issue typical in traditional recurrent neural networks, this approach enables more effective modeling of long-range dependencies. By integrating recursive inference with convolutional training mechanisms, SSMs effectively balance real-time processing efficiency and parallel computation, significantly improving computational performance. Furthermore, SSMs possess the ability to dynamically adapt their parameters, allowing for flexible alignment with the dynamic properties of diverse systems. SSM is widely applied in time–series analysis, control systems, and signal processing. Regarding RUL prediction models based on SSM, for example, one may refer to [21,22]. However, traditional SSMs face challenges with high computational complexity when processing long sequences. Mamba [23] is an improved state space model that was developed to overcome traditional SSMs’ limitations in modeling long sequences. It has linear complexity, allowing it to efficiently model long sequences while capturing their dynamic changes. It excels in tasks such as language modeling and time–series forecasting, while also offering high computational efficiency. Currently, the application of Mamba in RUL prediction is still relatively limited. For example, Liang and Zhao [24] proposed a Mamba-based state space model for early RUL prediction of lithium-ion batteries and demonstrated the method’s strengths in terms of prediction performance, robustness, and efficiency. Zhu et al. [25] integrated attention–Mamba networks with the Physics-Informed Neural Networks (PINNs) framework, incorporating hard-to-detect physical information into the neural network to improve the model’s RUL prediction accuracy.

Feature selection is a crucial step in modern data-driven modeling [26], focusing on identifying and extracting the most relevant features from the original feature set to reduce redundancy and retain essential information. This process is designed with the aim of enhancing model performance, interpretability, and computational efficiency. Causal discovery algorithms are designed to uncover causal relationships between variables from observational data. Studies on improving feature selection through causal structure learning can be found in works such as [27]. Transfer entropy (TE) is an information–theoretic metric used to measure the directional flow of information or causal influence between time–series. In the context of equipment life prediction, applying the TE algorithm helps identify and select key causal relationships from large datasets, leading to more accurate prediction models. Causal discovery has been widely applied in time–series forecasting, such as in [28,29], which demonstrated the effectiveness of this method in improving model performance.

Although the transfer entropy algorithm demonstrates excellent performance in causality identification, its computational complexity is considerable, particularly when handling high-dimensional time–series data [30], as the computational burden grows significantly. Additionally, the TE algorithm is sensitive to noise, and the presence of noise in the data may lead to erroneous causality inferences. Inspired by [5,31], this paper proposes an RUL prediction model based on Bidirectional Mamba (BiMamba) and causal discovery algorithms. Firstly, the impact of random noise is effectively reduced through exponential smoothing techniques. Subsequently, the maximum information transfer entropy method, as described in [31], is employed to construct a causal graph of feature variables; based on these, key feature variables are selected. Then, utilizing these key feature variables, a model (namely, Cau–BiMamba–LSTM) combining BiMamba, LSTM, and causality is applied for RUL prediction.

This paper makes the following key contributions:

(1) To tackle the noise issue in time–series data, exponential smoothing is employed to weight and average the data points. Furthermore, the maximum information transfer entropy algorithm is utilized to identify more accurate causal relationships. This causality-driven feature selection method enhances the interpretability and prediction accuracy of the model.
(2) This paper is the first to apply the BiMamba model to the RUL prediction of aircraft engines. By integrating a hybrid model that combines bidirectional processing mechanisms, Mamba, attention mechanisms, and LSTM, the ability to model long sequences is significantly improved. The approach takes full advantage of Mamba’s low complexity and high computational efficiency, achieving enhanced accuracy while minimizing computational resource usage.
(3) Our model demonstrates superior performance on the C-MAPSS dataset, highlighting its potential as a versatile method for predicting RUL. The Cau–BiMamba–LSTM model achieves optimal performance in terms of *RMSE* and *SCORE* on the C-MAPSS dataset, with a parameter count as low as 3323. The prediction accuracy on the FD002 and FD004 datasets outperforms most existing models. Specifically, on the most complex sub-dataset, FD004, the *RMSE* reaches 14.37 and *SCORE* reaches 948, making it the best-performing model in terms of prediction accuracy compared to all other models.

The sections of the paper are organized as follows: in Section 2, preliminaries are discussed; in Section 3, the proposed Cau–BiMamba–LSTM prediction framework is presented; in Section 4, the experimental setup and the efficiency of the approach are developed; and finally, Section 5 concludes the paper and proposes some further research directions.

## 2. Preliminaries

### 2.1. State Space Model and Mamba

The traditional state space model is used conventionally in control system theory to describe the dynamic behavior of systems and estimate states from observational data. It transforms the input, x(t)∈R, into the output, y(t)∈R, with the help of the hidden state, $h\left(t\right)\in R^{N\times 1}$ [32]:

$$h^{\prime }\left(t\right)=Ah\left(t\right)+Bx\left(t\right),$$

(1) y(t)=Ch(t).

where $h^{\prime }\left(t\right)$ is the derivative of the current state, $A\in R^{N\times N}$ is the state transition matrix, which describes how the state changes over time, $B\in R^{N\times 1}$ represents the matrix that defines the input’s impact on state changes, and $C\in R^{1\times N}$ is the output matrix.

To handle discrete rather than continuous data, the Zero-Order Hold technique [33] is employed, which converts the discretized signals into continuous signals suitable for the SSM. Specifically, a time-scale parameter Δ∈R is used to transform the continuous parameters A and B into their corresponding discrete parameters:

$$\bar{A}=exp\left(\Delta A\right),$$

(2) $$\bar{B}={\left(\Delta A\right)}^{-1}\left(exp\left(\Delta A\right)-I\right)\Delta B.$$

rephrased using the step size Δ as follows:

$$h\left(t\right)=\bar{A}h_{t-1}+\bar{B}x_{t},$$

(3) $$y_{t}=Ch_{t}.$$

In addition, the model can use global convolution calculations to produce the output:

$$\bar{K}=\left(C\bar{B},C\bar{A}\bar{B},\ldots ,C{\bar{A}}^{M-1}\bar{B}\right),$$

(4) $$y=x*\bar{K}.$$

Here, *M* represents the length of the input sequence, *x*; the convolution kernel is denoted by $\bar{K}\in R^{M}$. Clearly, the discrete SSM shares a similar structure to a recurrent neural network but offers superior parallel computing capabilities, which traditional RNNs, reliant on nonlinear activation functions, cannot achieve.

Traditional SSMs are time-invariant, meaning their A, B, C and Δ are independent of the model’s input variables. This limitation restricts the ability to model context effectively and impacts overall performance [34]. To address this issue, [23] proposed Mamba as a potential alternative. One of the core innovations of Mamba is the introduction of the time-varying selection mechanism. The state transition matrices are defined as follows:

(5) $$A_{t}=f_{A}\left(x\left(t\right)\right),\;B_{t}=f_{B}\left(x\left(t\right)\right)$$

**Figure 1:**
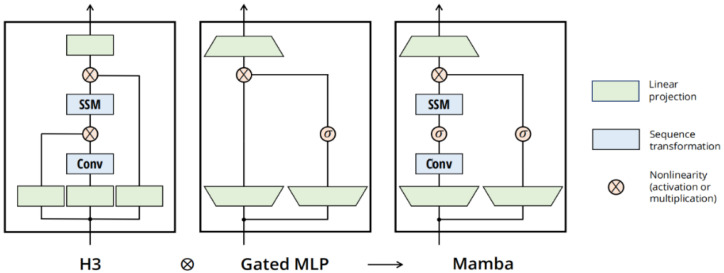
Graphical representation of Mamba Block [23].

The proposed mechanism dynamically adjusts the model’s weight matrices, enabling better adaptation to changes in the input sequence, leading to enhanced model performance. Moreover, Mamba introduces a hardware-aware algorithm that boosts computational efficiency through parallel scanning, kernel fusion, and the effective recomputation of intermediate results stored in memory. The structure of Mamba is depicted in Figure 1.

**Figure 2:**
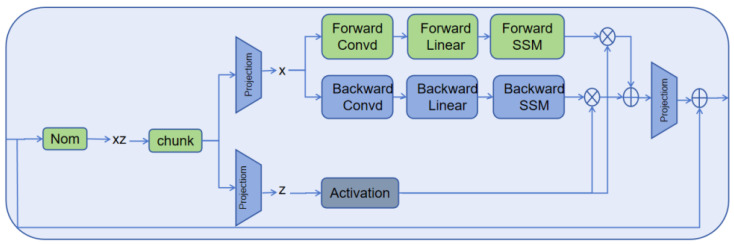
BiMamba module in [32].

The BiMamba architecture, shown in Figure 2 and introduced by Liu et al. [32], significantly enhances model performance by leveraging dual-branch attention mechanisms to improve feature extraction and fusion.

First, the input sequence $X_{l-1}$ is normalized by $X_{l-1}^{\prime }=Norm\left(X_{l-1}\right)$. Then, $X_{l-1}^{\prime }$ is linearly projected onto *x* and *z*: $x=Linear^{x}\left(X_{l-1}^{\prime }\right)$, $z=Linear^{z}\left(X_{l-1}^{\prime }\right)$. *x* is for subsequent state space modeling, and *z* is for the gating mechanism. Next, the sequence undergoes forward and backward processing. For the forward operation, *o*, after passing through a 1D convolutional layer and applying the SiLU activation function, we obtain $x_{o}^{\prime }$. Then, we compute the linear projection of the SSM parameters $B_{o}=Linear_{o}^{B}\left(x_{o}^{\prime }\right)$, $C_{o}=Linear_{o}^{C}\left(x_{o}^{\prime }\right)$ and the time-step parameter is calculated as $\Delta_{o}=log\left(1+exp\left(Linear_{o}^{\Delta }\left(x_{o}^{\prime }\right)\right)+Parameter\Delta_{o}\right)$, where $Parameter\Delta_{o}$ is learnable parameter. Subsequently, using ${\bar{A}}_{o}=\Delta_{o}\cdot Parameter\Delta_{o}$, ${\bar{B}}_{o}=\Delta_{o}\cdot B_{o}$, the system’s continuous-time representation is converted to a discrete-time representation, making the SSM more efficient for computation on a computer. The obtained matrices ${\bar{A}}_{o}$, ${\bar{B}}_{o}$, $C_{o}$, and $x_{o}^{\prime }$ are fed into the state space model to produce the output of the forward pass $y_{forward}$. The backward pass follows the same process, with the outputs from both the forward pass $y_{forward}$ and backward pass $y_{backward}$ being multiplied with *z* after applying the nonlinear SiLU activation to compute $y_{forward}^{\prime }$ and $y_{backward}^{\prime }$, respectively. Ultimately, the final output $X_{l}$ is derived using the equation $X_{l}=Linear^{T}\left(y_{forward}^{\prime }+y_{backward}^{\prime }\right)+X_{l-1}$.

### 2.2. Attention Mechanism

#### 2.2.1. Self-Attention Mechanism

The self-attention mechanism is a technique used to capture dependencies between elements in sequence data, initially introduced by Vaswani et al. in 2017 [35], and has become a fundamental part of the transformer model. The self-attention mechanism dynamically assigns attention weights by calculating the relevance of each element in the sequence to other elements, thereby better capturing global dependencies. Let the input be $X\in R^{N\times d}$, where *N* is the sequence length and *d* is the feature dimension. The definitions of query (denoted as *Q*), key (denoted as *K*) and value (denoted as *V*) are as follows:

(6) $$Q=XW_{Q},\;K=XW_{K},\;V=XW_{V}$$

where $W_{Q}\in R^{d\times d_{k}},W_{K}\in R^{d\times d_{k}},W_{V}\in R^{d\times d_{k}}$ are the weight matrices. The attention weights are:

(7) $$Attention\left(Q,K,V\right)=softmax\left(\frac{QK^{T}}{\sqrt{d_{k}}}\right)$$

The output is:

(8) output=Attention(Q,K,V)·V

The self-attention mechanism captures long-range dependencies by transforming the input data into matrix format. It aggregates the input features with weighted attention, emphasizing key information while suppressing details that are less relevant to the current task, effectively capturing long-distance dependencies and global information.

#### 2.2.2. Additive Attention Mechanism

The additive attention mechanism, first introduced by [36], is a commonly used attention technique. Compared to the traditional dot product attention, the additive attention mechanism offers a more stable computational process, particularly when handling high-dimensional data. It effectively mitigates potential numerical issues that may arise in the dot product method. For each query–key pair (Q,K), the alignment *SCORE* is computed using the tanh activation function:

(9) $$e\left(Q,K\right)=V^{T}tanh\left(W_{Q}+W_{K}K+b\right)$$

Attention weights are:

(10) $$\alpha \left(Q,K\right)=\frac{e(Q,K)}{\sum_{K^{\prime }}e\left(Q,K^{\prime }\right)}$$

The output is a weighted sum of all value vectors *V*.

### 2.3. Exponential Smoothing

Time–series data often contain various random fluctuations or noise, which may not be related to the true underlying trend. Exponential smoothing, through weighted averaging, enables the model to focus more on recent genuine changes, thereby effectively reducing noise [37]. The goal is to smooth the data and minimize the interference of random fluctuations on the model. Its mathematical expression is:

(11) $${\hat{x}}_{t}=\frac{\sum_{i=0}^{t}\omega_{i}x_{t-i}}{\sum_{i=0}^{t}\omega_{i}}$$

There are various exponential smoothing methods, and the definition of the smoothing coefficient $w_{i}$ can vary; for example, ref. [5] uses the following method: $\omega_{i}={\left(1-\gamma \right)}^{i},\gamma =\frac{2}{\left(1+s\right)}$. For example, the formula for simple exponential smoothing is as follows:

(12) $${\hat{x}}_{t}=\alpha \cdot x_{t}+\left(1-\alpha \right)\cdot x_{t-1}$$

When α is close to 1, $x_{t}$ represents the actual observed value, and $\hat{x_{t}}$ is the smoothed value. The model becomes more sensitive to the most recent observations, with a weaker smoothing effect on historical data, making it suitable for situations where the data changes rapidly. When α is close to 0, the model places more emphasis on smoothing the historical data, making it more suitable for situations where the data changes slowly.

### 2.4. Maximum Information Transfer Entropy

Information entropy can be utilized to measure causal relationships. Assuming that the information transfer between two variables will reduce the uncertainty of the system, it is termed as transfer entropy [38]. When the transfer entropy from X to Y is greater than from Y to X, we designate X as the cause and Y as the effect, thus establishing a causal relationship between the two variables. Causal network graphs utilize nodes to represent variables and edges to illustrate the causal connections between them. This graphical model enables us to understand the interactions and mutual influences among various components of mechanical equipment more clearly, providing an important foundation of accuracy for constructing predictive models.

Transfer entropy (TE), introduced by Schreiber in 2000 [38], quantifies the directed information flow or causal influence between two stochastic processes or time–series. maximum information transfer entropy (MITE) further optimizes the calculation of transfer entropy to capture the strongest information transfer relationships between systems. In reference [31], researchers introduced a causal modeling approach utilizing maximum information transfer entropy (MITE-CM) for analyzing causality in industrial control systems. This method combines transfer entropy with the Maximum Information Coefficient (MIC) network to measure causal interactions within systems. For more comprehensive information on the MITE-CM technique, consult references [29,31].

## 3. Methodology

**Figure 3:**
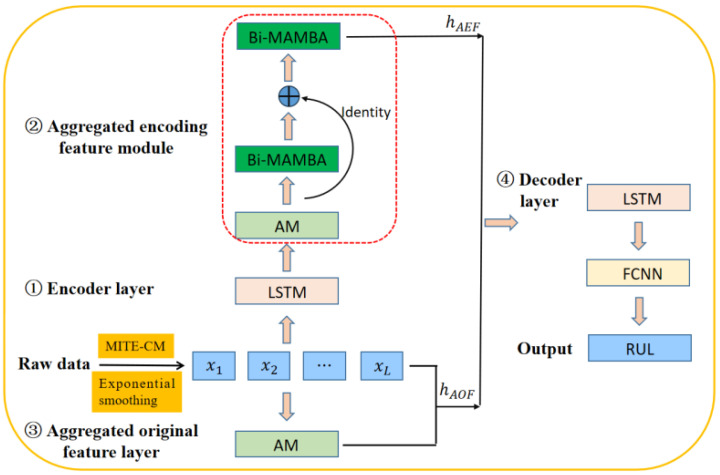
Structure of the Cau–BiMamba–LSTM framework.

The authors of [29] employ the MITE-CM algorithm to perform causal analysis on the CMAPSS dataset for aircraft engines, thereby improving the performance of prediction. However, they do not consider the impact of noise in the data on the causal relationships, which leads to inaccurate causal inference. To address this limitation, we utilize an approach that combines exponential smoothing with the MITE-CM algorithm for causal feature selection and introduce a new BiMamba module, integrating models such as LSTM and attention mechanisms for RUL prediction. The network architecture of our proposed Cau–BiMamba–LSTM model is illustrated in Figure 3.

First, this paper employs the simple exponential smoothing method and the MITE-CM algorithm for causal feature selection. Subsequently, the selected features are processed through an encoder layer, an aggregated encoding feature (AEF) module, an aggregated original feature (AOF) layer, and a decoder layer for RUL prediction. Compared to the methods proposed in [5,29], our innovation lies in the design of a BiMamba-based aggregated encoding feature layer. By leveraging a BiMamba module combined with residual networks, our approach enables the more precise extraction of data features. The architecture of the BiMamba module, as described in [32], is depicted in Figure 2.

We utilize an self-attention mechanism to aggregate the original features of key variables, employ LSTM for encoding, and leverage a combination of BiMamba, residual connection, and additive mechanisms for aggregating the encoded features. The two types of aggregated features are then concatenated and decoded using LSTM. The LSTM output is processed by a fully connected layer to produce the final decoded result, thereby achieving RUL prediction. In the BiMamba-based aggregated encoding feature layer, a self-attention mechanism is initially applied, with its output acting as the input for the initial BiMamba module. The output from this module is then combined with the self-attention mechanism output through a residual connection and passed into the second BiMamba module. This design effectively harnesses the capabilities of BiMamba, allowing it to capture a wide range of features and produce a more detailed representation, which enhances the accuracy of RUL prediction.

The Cau–BiMamba–LSTM model has advantages in the following three aspects:

(1) Information flow perspective: BiMamba and LSTM exhibit complementarity. BiMamba excels at capturing long-range dependencies and effectively modeling complex long-term trends in time–series data [32]. On the other hand, LSTM is better suited for local pattern recognition, as it can remember and forget specific information within shorter time spans. By combining these two models, the hybrid model leverages their respective strengths, capturing both long-term trends and short-term fluctuations. This complementarity enhances the model’s performance when handling complex time–series data.
(2) Computational complexity: The computational complexity of the Cau–BiMamba–LSTM model remains linear [5,32]. When dealing with large-scale datasets, integrating multiple models can significantly improve performance. However, the addition of models often results in increased computational burden. Through optimized design, the proposed hybrid model maintains high performance without a significant increase in computational cost, ensuring that the complexity grows linearly.
(3) Innovative causal feature selection and effective fusion with attention mechanism: The hybrid model innovatively combines causal feature selection with an attention mechanism. It fully leverages the advantages of transfer entropy theory for feature selection. The model uses exponential smoothing to remove noise and employs maximum transfer entropy for causal feature selection to enhance subsequent prediction accuracy. Additionally, the attention mechanism is incorporated to dynamically focus on important features. This allows the model to automatically prioritize features that contribute more significantly to the prediction, achieving effective fusion of information. By combining transfer entropy for causal feature selection with attention mechanisms for feature weighting, the hybrid model efficiently utilizes input features and improves overall prediction performance.

In summary, the Cau–BiMamba–LSTM model integrates various independent yet complementary modules, including exponential smoothing, causal feature selection, BiMamba, LSTM, and attention mechanisms. This combination not only fully leverages the strengths of each module but also overcomes the limitations that individual modules may have. Exponential smoothing reduces the impact of noise, the MITE-CM algorithm enhances causal inference capabilities, and the BiMamba module, through the integration of LSTM and the attention mechanism, improves feature learning and modeling of temporal dependencies. As a result, the model effectively improves the accuracy and reliability of RUL prediction by comprehensively addressing noise suppression, causal relationship discovery, and the learning of both long-term and short-term features in time–series.

## 4. Experimental Procedure and Analysis

### 4.1. Dataset

The C-MAPSS dataset [39], created by NASA, simulates real turbofan engines. It serves as an open-access dataset for studies in health monitoring and RUL prediction. The dataset includes four subsets, each representing different operating conditions and fault scenarios. The training set contains sampled values of various state parameters taken at different time points throughout a complete cycle, ranging from normal operation to failure. In contrast, the test set contains state parameters at a specific time point just before failure, along with the corresponding remaining lifespan. The dataset comprises 26 columns: the initial column identifies the engine ID, followed by the current operational cycle in the second column. The subsequent three columns outline the operating conditions, while columns 6 through 26 provide numerical data from 21 sensor readings. Table 1 offers a comprehensive overview of the C-MAPSS dataset.

The C-MAPSS dataset consists of four sub-datasets (FD001-FD004), with increasing complexity: FD001 contains a single operational condition and fault mode, while FD004 includes six operational conditions and two composite fault modes. Each sub-dataset’s training set contains full lifetime data from 100 to 249 engines, while the test set contains partial data from 100 to 248 engines. The challenges in processing the dataset mainly lie in operational condition shifts, noise interference, and the complex coupling of fault characteristics. For instance, the standard deviation of sensor noise in FD003 reaches 0.5%, and FD002 covers six operational conditions that span flight altitudes from sea level to 35,000 feet. These characteristics make the dataset an important benchmark for evaluating the robustness of predictive models, particularly in terms of handling multimodal degradation trajectories and generalization capabilities, and offer significant research value.

### 4.2. Evaluation Criteria

The model’s performance is evaluated using two metrics: Root Mean Square Error (*RMSE*) and the *SCORE* function. Among these, *RMSE* is a standard tool for measuring the prediction accuracy of regression models. Given a set of true values $y_{1},\;y_{2},\;\ldots ,\;y_{N}$ and the corresponding model predictions ${\hat{y}}_{1},\;{\hat{y}}_{2},\;\ldots ,\;{\hat{y}}_{N}$, *N* represents the total count of data points. Apply Equation (13) to calculate the *RMSE*. A lower *RMSE* value reflects improved model prediction accuracy. The definition of the *SCORE* function can be found in (14), where *d* represents the deviation between the predicted RUL and the actual RUL, defined as $d_{i}=\hat{y_{i}}-y_{i}$. Since early fault detection is crucial for engines, when *d* < 0, it suggests the predicted RUL underestimates the actual RUL, implying there is still time for maintenance, and thus the penalty is smaller. Conversely, when *d* > 0, it indicates the predicted RUL overestimates the true RUL, which could lead to machine failure without timely repair, resulting in a larger penalty.

(13) $$RMSE=\sqrt{\frac{1}{N}\sum_{i=1}^{N}{\left({\hat{y}}_{i}-y_{i}\right)}^{2}}$$

(14) $$SCORE=\sqrt{\frac{1}{N}\sum_{i=1}^{N}S_{i}},\;S_{i}=\left\{\begin{array}{c} e^{-\frac{d_{i}}{13}}-1,\;\mathrm{for}\;d_{i}<0\\ e^{\frac{d_{i}}{10}}-1,\;\mathrm{for}\;d_{i}\geq 0 \end{array}\right.$$

### 4.3. Experimental Setup

All experiments are implemented on a PC with 13th Gen Intel(R) Core(TM) i9-13900HX GPU (2.20 GHz) with 32-GB RAM, and the programming platform was python 3.10.

In the causal algorithm based on simple exponential smoothing and the MITE-CM algorithm, the smoothing factor α is set to 0.3. In the encoder layer, a two-layer LSTM with six hidden units is used. The AEF module employs an additive mechanism for the attention scoring function, whereas the AOF layer adopts a concatenation-based mechanism. Table 2 displays the smoothing rate values in the exponential smoothing method during data preprocessing, the hidden units and attention size in the AEF module, along with the attention size in the AOF layer. The decoder layer uses a two-layer LSTM, each with six hidden units and a fully connected layer also containing six hidden units. These parameters were obtained through experimental tuning. During the training process, the batch size was set to 128, and the number of epochs was set to 40. During the training process, the batch size was set to 128, and the number of epochs was set to 40.

We have provided the grid search validation results for the smooth factor (s value) and the validation results for the number of hidden units in the AEF module, which are presented in Table 3 and Table 4. These also serve as the basis for the parameter settings in Table 2.

## 5. Results and Analysis

### 5.1. Causality-Driven Feature Selection

**Figure 4:**
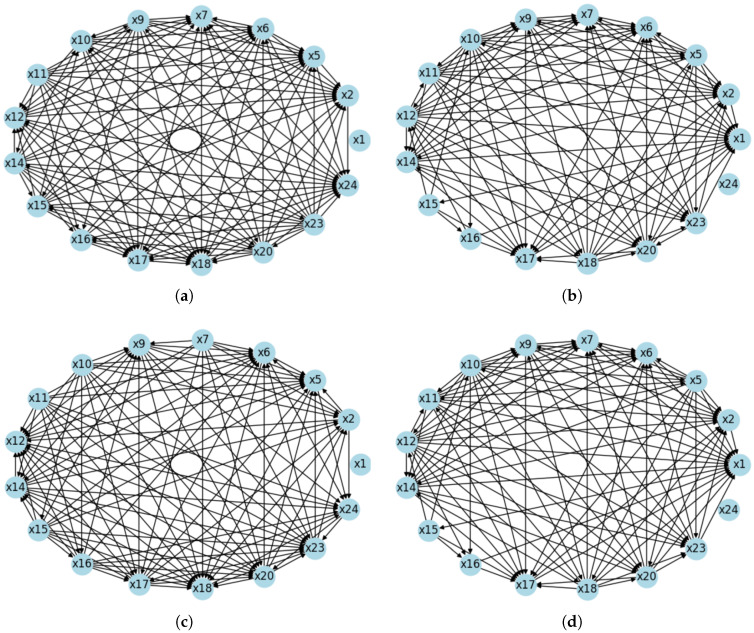
Causal network graphs on C-MAPSS dataset. The causal relationship diagrams for FD001, FD002, FD003, and FD004 are represented by (**a**–**d**), respectively.

Using the causal algorithm based on simple exponential smoothing and the MITE-CM algorithm, the resulting causal graph is shown in Figure 4. These graphs differ from the results presented in the literature [29].

From Figure 4, we select the key feature variables, as shown in Table 5.

### 5.2. RUL Prediction Result

**Figure 5:**
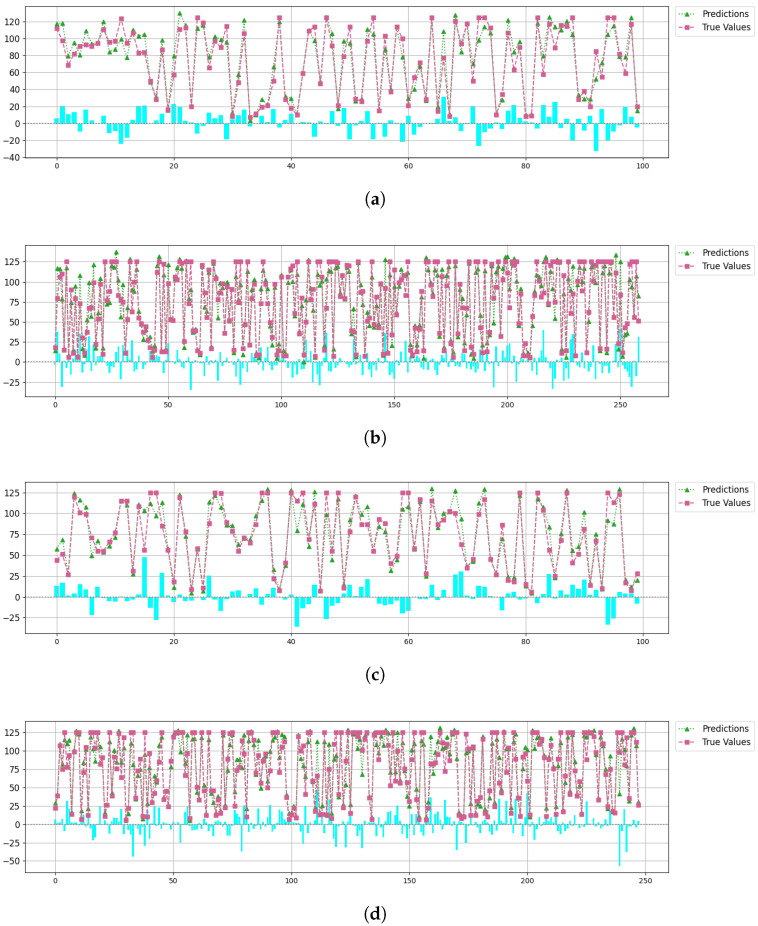
Prediction results of Cau–BiMamba–LSTM; (**a**–**d**) represent the comparison results of the true values and predicted values for FD001, FD002, FD003, and FD004, respectively. The blue bars represent the prediction bias for each engine in each sub-dataset.

Figure 5 compares the predicted and true RUL values for each engine across the four data subsets. The chart displays predicted and actual values for various engines, organized by engine ID. The blue line represents the predicted RUL values of the engines, while the orange line represents the true RUL values. It is evident that the proposed prediction framework achieves highly accurate results. In Figure 5, we not only list the actual RUL values and predicted RUL values for each engine, but also provide the deviation values, which are visualized using bar charts to show the prediction bias for each sub-dataset.

We evaluated the proposed approach against other established RUL prediction methods which are based on attention, LSTM, or Mamba, including GCU-Transformer [40], BIGRU-TSAM [41], DA-Transformer [15], GA-Transformer [42], CNN-BiLSTM-3DAttention [19], DA-LSTM [5], Cau-DA-LSTM [29], Mamba-PINN [25], and ABiTCI [43]. The results of the comparison are presented in Table 6, with optimal results in bold and suboptimal results underlined. The same format is applied to Table 7, Table 8 and Table 9 for consistency. As shown in Table 7, the Cau–BiMamba–LSTM model achieves the lowest mean and variance in *RMSE*, along with the lowest mean and the second-lowest variance in *SCORE*. This indicates that our proposed method delivers the best overall performance across the four subsets. Furthermore, the lower variance suggests that the exponential smoothing-based model exhibits strong generalization capability and robustness.

**Figure 6:**
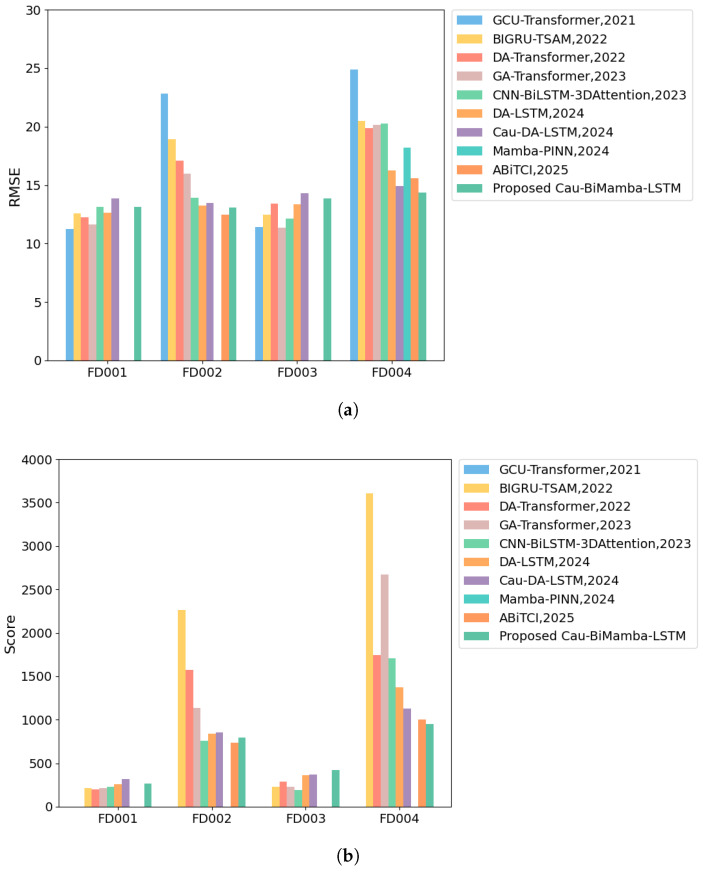
Visualization of model performance comparison. (**a**) Comparison of *RMSE* for the ten models; (**b**) comparison of *SCORE*.

As demonstrated in Table 6 and Figure 6, the Cau–BiMamba–LSTM model achieves outstanding performance on both the FD004 and FD002 datasets. On the FD004 dataset, which is the most challenging subset of C-MAPSS, the BiMamba-DA-LSTM model demonstrates superior performance in both *RMSE* and *SCORE* compared to other models. This indicates that the model has a strong capability in handling intricate data patterns and long-term dependencies, enhancing its suitability for industrial applications, such as fault detection. On the FD002 dataset, the Cau–BiMamba–LSTM model’s *RMSE* is marginally higher than the optimal model but remains at a suboptimal level. The introduction of causal discovery algorithms to extract key features and the incorporation of the BiMamba module enable the model to efficiently capture the complex dependencies in the equipment degradation process, showing good generalization ability across different datasets and outperforming most comparison models overall. In conclusion, the Cau–BiMamba–LSTM model demonstrates excellent performance in RUL prediction tasks. Its efficiency, accuracy, and lightweight design will make it highly promising for practical industrial applications.

Table 8 shows that the proposed Cau–BiMamba–LSTM has a lower number of parameters and moderate computational efficiency, making it a lightweight RUL prediction model. This also demonstrates that the integration of the Mamba model with the LSTM model does not significantly increase computational complexity.

The essential difference between FD002/FD004 and FD001/FD003 lies in the coupling of multi-operational conditions and complex fault modes. The fault degradation trajectories of FD002/FD004 are disturbed by multi-operational conditions, and the fault evolution process is influenced by the coupling of multiple physical fields, resulting in stronger nonlinearity and randomness. Our model suppresses such disturbances through noise-robust mechanisms and explicitly captures the complex dependencies during condition switching through bidirectional state space modeling and a condition-aware mechanism. In contrast, the single-operational condition environment of FD001 and FD003 means that such complex modeling could introduce unnecessary computational overhead, thereby affecting performance. The benchmark model has been widely validated for its mature performance in simpler scenarios like FD001/FD003, but it shows limitations in predictive stability in multi-operational, complex environments. Our model, through a structured design, balances the modeling capability and computational efficiency for complex scenarios. Its core objective is to enhance the reliability of multi-condition coupled fault prediction, which is more common in practical engineering, thus better aligning with the key needs in the field of industrial health state prediction. Moreover, compared to existing models, it achieves optimal average generalization performance across all data distributions.

### 5.3. Ablation Study

The ablation study compares the performance of different model configurations using *RMSE* and *SCORE* metrics on the C-MAPSS dataset. The results show that the proposed Cau–BiMamba–LSTM consistently achieves the best performance, with the lowest *RMSE* and *SCORE* values in all datasets, demonstrating its effectiveness. Table 9 demonstrates that the ability to model long sequences is crucial, the MITE-CM algorithm for causal feature selection is effective, and exponential smoothing for noise reduction is necessary. This confirms the necessity of each core module.

## 6. Conclusions

This study proposes a lightweight Cau–BiMamba–LSTM model, enhancing RUL prediction accuracy and robustness. By integrating causal algorithms for feature selection, the BiMamba module for efficient sequence modeling, and the attention module for feature extraction, the model achieves notable performance improvements on the C-MAPSS dataset. Experimental results demonstrate that the proposed model reduces the *RMSE* to 14.37 and the *SCORE* to 948 on the C-MAPSS FD004 dataset, surpassing existing models (based on attention, LSTM, or Mamba) on the FD004 dataset, while also achieving a near-optimal level on the FD002 dataset. This validates the model’s generalization ability across multiple datasets, particularly its excellent performance under complex data distributions and long sequence dependencies. Furthermore, the model’s lightweight design enables deployment on resource-limited edge devices, meeting the demands of real-time prediction. Existing pure-data-driven RUL prediction models often face challenges in balancing prediction accuracy, computational complexity, and interpretability. The proposed model addresses these challenges by incorporating feature selection interpretability, making it both lightweight and highly accurate. It shows enhanced stability and robustness when dealing with diverse operating conditions and complex time–series data.

The model still has some limitations: While the attention mechanism can highlight the importance of different features in the prediction, it does not provide clear physical or system-level explanations, leading to a lack of interpretability in the model’s decision-making process. Additionally, it does not consider physical constraints and lacks effective integration of physical knowledge, which limits the model’s generalization ability to some extent. Future work will focus on integrating multi-source data, including time–series and causal graph data. We aim to combine Mamba with physics-informed networks to build an interpretable prediction framework. Additionally, we plan to consider integrating data-driven models with physical models to construct a hybrid prediction framework, further enhancing prediction performance and interpretability.

## Figures and Tables

**Table 1:** Overview of the C-MAPSS dataset.

Sub-Dataset	C-MAPSS			
	FD001	FD002	FD003	FD004
Training engines	100	260	100	249
Testing engines	100	259	100	248
Operating conditions	1	6	1	6
Failure mode	1	1	2	2

**Table 2:** Hyperparameter settings in datasets.

	FD001	FD002	FD003	FD004
Hidden units number in AEF module	8	8	10	6
Attention size in AEF module	1	1	3	3
Attention size in AOF layer	16	16	6	6
Smooth rate ( s value)	25	30	30	30

**Table 3:** Sensitivity of hidden units number in AEF module.

Hidden Units Number in AEF Module	*RMSE*				*SCORE*			
	FD001	FD002	FD003	FD004	FD001	FD002	FD003	FD004
2	15.30	18.76	22.59	22.82	405	1845	3356	6030
4	14.55	13.79	15.94	15.38	383	862	747	1171
6	14.15	13.50	14.64	14.37	391	900	546	**948**
8	13.16	13.09	13.73	15.26	**264**	**793**	451	1180
10	13.04	13.44	13.85	14.55	272	858	**426**	1022

**Table 4:** Sensitivity of smooth rate (s value).

Smooth Rate (s value)	*RMSE*				*SCORE*			
	FD001	FD002	FD003	FD004	FD001	FD002	FD003	FD004
20	14.15	14.44	14.72	14.78	330	1020	580	958
25	**13.16**	13.56	14.35	14.59	**264**	821	519	990
30	13.49	**13.09**	**13.85**	**14.37**	290	**793**	**426**	**948**
35	13.98	13.53	14.48	14.58	319	889	465	1035

**Table 5:** Variables utilized in the proposed Cau–BiMamba–LSTM model.

	FD001 and FD003	FD002 and FD004
Variable name	ID	ID
Sensor signal	2, 3, 4, 6, 7, 8, 9, 11, 12, 13, 14, 15, 17, 20, 21	2, 3, 4, 6, 7, 8, 9, 11, 12, 13, 14, 15, 17, 20
Operational setting	2	1, 2

**Table 6:** Model performance comparison on the C-MAPSS dataset.

Methods	*RMSE*				*SCORE*			
	FD001	FD002	FD003	FD004	FD001	FD002	FD003	FD004
GCU-Transformer [40], 2021	**11.27**	22.81	11.42	24.86	N/A	N/A	N/A	N/A
BIGRU-TSAM [41], 2022	12.56	18.94	12.45	20.47	213	2264	233	3610
DA-Transformer [15], 2022	12.25	17.08	13.39	19.86	**198**	1575	290	1741
GA-Transformer [42], 2023	11.63	15.99	**11.35**	20.15	215	1133	228	2672
CNN-BiLSTM-3DAttention [19], 2023	13.12	13.93	12.15	20.24	231	760	**196**	1710
DA-LSTM [5], 2024	12.62	13.22	13.34	16.25	263	842	360	1372
Cau-DA-LSTM [29], 2024	13.87	13.45	14.31	14.93	321	853	369	1129
Mamba-PINN [25], 2024	N/A	N/A	N/A	18.18	N/A	N/A	N/A	N/A
ABiTCI [43], 2025	N/A	**12.46**	N/A	15.57	N/A	**736**	N/A	1003
Proposed Cau–BiMamba–LSTM	13.16	13.09	13.85	**14.37**	264	793	426	**948**

**Table 7:** Mean and variance of different models.

Methods	*RMSE*		*SCORE*	
	μ (*RMSE*)	σ (*RMSE*)	μ (*SCORE*)	σ (*SCORE*)
GCU-Transformer [40], 2021	17.59	6.96	N/A	N/A
BIGRU-TSAM [41], 2022	16.11	4.81	1530.00	1522.13
DA-Transformer [15], 2022	15.65	3.30	951.00	764.03
GA-Transformer [42], 2023	14.78	3.96	1062.00	1103.01
CNN-BiLSTM-3DAttention [19], 2023	14.86	3.78	724.25	738.03
DA-LSTM [5], 2024	13.86	1.51	709.25	476.27
Cau-DA-LSTM [29], 2024	14.14	0.67	667.50	370.42
Mamba-PINN [25], 2024	18.18 *	N/A *	N/A	N/A
ABiTCI [43], 2025	14.02 **	1.45 **	869.50 **	133.50 **
Proposed Cau–BiMamba–LSTM	**13.62**	**0.47**	**607.75**	288.50

The asterisk (*) is used to annotate data calculation methods that require special explanation. A single asterisk () * indicates that the model has only one column of *RMSE* or *SCORE* data. Double asterisks () ** indicate that the model has only two columns of *RMSE* or *SCORE* data.

**Table 8:** Comparison for computation quantity.

Model	Proposed	DA-LSTM	Cau-DA-LSTM	CNN-BiLSTM-3DAttention	DA-Transformer	BIGRU-TSAM	GCU-Transformer
	Cau–BiMamba–LSTM	[5]	[29]	[19]	[15]	[41]	[40]
Parameter num	**3323**	3550	3898	151,900	116,591	2,825,443	399,700
FLOPs	$2.31\times 10^{6}$	$1.27\times 10^{5}$	$6.39\times 10^{6}$	$1.70\times 10^{5}$	$7.44\times 10^{6}$	$1.68\times 10^{8}$	$3.93\times 10^{5}$

**Table 9:** Ablation study.

Methods	*RMSE*				*SCORE*			
	FD001	FD002	FD003	FD004	FD001	FD002	FD003	FD004
No BiMamba in AEF module	14.22	13.18	14.68	14.54	359	810	547	987
No causal feature selection	13.45	13.45	14.24	17.80	333	972	621	2158
Causal feature selection without exponential smoothing	14.53	13.56	14.60	18.67	416	958	575	2611
Proposed Cau–BiMamba–LSTM	**13.16**	**13.09**	**13.85**	**14.37**	**264**	**793**	**426**	**948**

## Data Availability

The C-MAPSS dataset is a U.S. Government Work in the public domain, hosted by NASA’s Prognostics Center of Excellence (PCoE) with open access for non-commercial research. Our manuscript has fully complied with NASA’s terms through the following measures: (1) proper citation of the original technical report (Saxena et al., 2008 [39]); (2) exclusive use of the data for academic research purposes. The C-MAPSS dataset used in this study is publicly available from NASA’s Prognostics Data Repository (https://ti.arc.nasa.gov/tech/dash/groups/pcoe/prognostic-data-repository/) under the U.S. Government Work policy.
